# Phenotypic and genotypic virulence features of staphylococcal strains isolated from difficult-to-treat skin and soft tissue infections

**DOI:** 10.1371/journal.pone.0246478

**Published:** 2021-02-02

**Authors:** Mădălina Preda, Mara Mădălina Mihai, Laura Ioana Popa, Lia-Mara Dițu, Alina Maria Holban, Loredana Sabina Cornelia Manolescu, Gabriela-Loredana Popa, Andrei-Alexandru Muntean, Irina Gheorghe, Carmen Mariana Chifiriuc, Mircea-Ioan Popa

**Affiliations:** 1 Department of Microbiology, Parasitology and Virology, Faculty of Midwives and Nursing, ‘Carol Davila’ University of Medicine and Pharmacy, Bucharest, Romania; 2 ‘Cantacuzino’ National Medico-Military Research and Development Institute, Bucharest, Romania; 3 Department of Oncologic Dermatology, ‘Carol Davila’ University of Medicine and Pharmacy, Bucharest, Romania; 4 Department of Dermatology, ‘Elias’ University Emergency Hospital, Bucharest, Romania; 5 Department of Bioinformatics, The National Institute of Research and Development for Biological Sciences, Bucharest, Romania; 6 Research Institute of the University of Bucharest (ICUB), Bucharest, Romania; 7 Department of Microbiology, Faculty of Biology, University of Bucharest, Bucharest, Romania; 8 Department of Microbiology, Faculty of Dental Medicine, ‘Carol Davila’ University of Medicine and Pharmacy, Bucharest, Romania; 9 Department of Microbiology, Faculty of Medicine, ‘Carol Davila’ University of Medicine and Pharmacy, Bucharest, Romania; Mississippi State University, UNITED STATES

## Abstract

Chronic infections represent an important burden on the healthcare system and have a significant impact on the patients’ quality of life. While *Staphylococcus* spp. are commensal bacteria, they can become pathogenic, leading to various types of infections. In this study we aimed to characterize the virulence profiles of staphylococcal strains involved in difficult-to-treat skin and soft tissue infections, from both phenotypic and genotypic points of view. Phenotypic ability of the strains to secrete soluble virulence factors was assessed by a culturing dependent assay and their capacity to develop biofilms on inert substrate was screened by an adapted crystal violet microtiter method. We also tested the presence of several virulence genes by PCR. Most of the studied strains were isolated from purulent secretions of acne lesions and frequently secreted two or three soluble virulence factors. Most frequently secreted soluble virulence factors were caseinase (89%), lipase (71%) and lecithinase (67%). Almost half of the strains produced a well-represented biofilm. The molecular characterization showed the presence of the genes *cna*, *hlg*, *clfA*, and *clfB*. Staphylococcal strains that produce difficult-to-treat skin and soft tissue infections seem to be characterized by an enhanced ability to produce different soluble virulence factors and to develop biofilms *in vitro*. Further studies need to be developed in other *Staphylococcus* spp. infections in order to confirm this hypothesis.

## 1. Introduction

*Staphylococcus* spp. are generally considered commensal bacteria [1, 2]. However, under immunosuppression or other conditions, they can produce skin and soft tissue, acute or chronic infections, osteomyelitis, pneumonia, endocarditis, implantable device associated infections, and other diseases [3]. Over the last few decades, staphylococcal resistance to antibiotics has emerged as an important issue, leading to difficult-to-treat infections and a significant burden to the healthcare system worldwide.

*S*. *aureus* is one of the most important microorganisms involved in nosocomial and community-acquired infections [4]. Although *S*. *aureus* can infect any tissue of the body, the primary site is most often represented by a breach in the skin [5]. *S*. *aureus* is responsible for the majority of bacterial cutaneous infections, such as surgical site infections, purulent cellulitis, cutaneous abscesses and many others [2, 6]. More recently, it was observed that cutaneous infections with community- acquired methicillin-resistant *S*. *aureus* (MRSA) have a tendency to outnumber the hospital-acquired ones [6], increasing the importance of the development of effective therapeutic alternatives [7]. Novel strategies represented by vaccines, nanoparticle delivery systems, bioactive antimicrobial materials, drugs targeting microbial virulence, bacterial metabolic activity or other pathogenic mechanisms might represent viable options of treatment [8–13]. The outcome of such approaches is correlated with bacterial virulence and resistance features [14].

The ability of *Staphylococcus* spp. to produce acute and chronic infections is due to multiple and redundant pathogenic determinants [15].

The development of staphylococcal biofilms is related to an increased tolerance to antibiotics as well as to the host’s immune defense mechanisms [13, 16]. The adhesion to organic and inorganic surfaces represents the first step in the development of staphylococcal biofilms, achieved through molecules such as teichoic acid, an important component of the bacterial cell wall [17], staphylococcal protein A, fibronectin binding proteins A and B, and clumping factors that can interact with the host’s cytokeratin, fibrinogen, fibronectin or loricin [18]. Biofilm formation can reduce the clearance of bacteria by the host’s neutrophils and macrophages, can facilitate macrophage cytotoxicity, and promote the apoptosis of keratinocytes [19]. Moreover, *S*. *aureus* can produce factors like extracellular fibrinogen-binding protein, extracellular complement-binding, and complement 4 binding protein that can inhibit C3b-mediated opsonization and ensue complement-mediated phagocytosis [4]. Askarian *et al*., 2016, demonstrated the role of Desmoglein 1 as host ligand for serine aspartate repeat containing protein D of *S*. *aureus* [20].

In addition to the ability to develop biofilms, *S*. *aureus* also secretes soluble virulence factors, that can disrupt epithelial barriers, promote immune evasion, and augment inflammation [21], increasing the importance of effective therapies. *Staphylococcus* spp. produce cytolytic exotoxins that form small pores in the plasma membrane, leading to the lysis of the host`s targeted cell [15]. The most well-known cytolytic toxins are the leucocidins, including Panton-Valentine leucocidin [15]. Another exotoxin produced by *S*. *aureus*, α-haemolysin, has the ability to disrupt cell-matrix adhesion [22]. Moreover, exogenous proteases of *S*. *aureus* can facilitate deeper tissue penetration of environmental antigens [19].

Besides the pathogenic implication of *S*. *aureus*, coagulase negative staphylococci species (CoNS) also influence the skin microbiome homeostasis as well as the skin barrier integrity [1–3]. It is assumed that the interaction between *S*. *aureus* and CoNS by quorum sensing modulates the virulence of each bacterial species, host-microbiome interactions, as well as immune responses [3]. CoNS, like *S*. *epidermidis*, are able to inhibit the development of *S*. *aureus* biofilms [19]. Moreover, CoNS can produce autoinducing peptides that inhibit the expression of *S*. *aureus* virulence factors and, consequently, diminish epithelial damage [3].

Despite their commensal status, increasing evidence have changed the awareness of clinicians and microbiologists regarding the virulence capacity of CoNS over the last years, as they became more and more associated with pathogenic processes, especially in the presence of other risk factors, such as immunosuppression, long-term hospitalization or the use of medical devices (catheters, prosthetic joints, and others) [6]. The most frequently involved species are: *S*. *epidermidis*, *S*. *haemolyticus*, *S*. *saprophyticus*, *S*. *capitis*, and *S*. *lugdunensis* [22]. Similar to *S*. *aureus*, CoNS are able to develop biofilms (up to 71.8% of isolates), exhibit an increased tolerance to antimicrobials and immune defense mechanisms, particularly *S*. *hominis* and *S*. *haemolyticus* strains [6, 15].

Thus, factors leading to staphylococcal infections are both host- and bacterial cell-dependent, the fate of infection being influenced by bacterial characteristics (such as bacterial tolerance and resistance to antimicrobials or the production of different virulence factors that promote bacterial adherence and/or invasion) and host defense mechanisms [23]. All these characteristics favor the survival and persistence of bacteria in hostile environments and lead to the development of difficult to treat infections with a long and sometimes severe evolution [15, 24].

The aim of our study was to identify the staphylococcal species involved in difficult-to-treat skin and soft tissue infections, and to establish their main virulence factors that could correlate with the severity of the infection. We focused on their ability to develop *in vitro* biofilms, to secrete soluble virulence factors, and to detect the presence of relevant virulence coding genes, which are known for their impact in the outcome of the infection.

## 2. Materials and methods

We selected one hundred strains of *Staphylococcus* spp. isolated from patients with difficult to treat infections (severe acne vulgaris, folliculitis decalvans, hidradenitis suppurativa, chronic surgical wounds, and other skin and soft tissue infections) between June 2019 and December 2019 in the ‘Cantacuzino’ National Medico-Military Institute for Research and Development from Bucharest. The study was approved by the ethics committee of the ‘Carol Davila’ University of Medicine and Pharmacy. A written consent was obtained from the patients included in the study. The experiments were performed in the laboratories of the ‘Cantacuzino’ National Medico-Military Institute for Research and Development and the University of Bucharest, Faculty of Biology. The identity of the bacterial strains was confirmed using classical microbiological phenotypic methods and MALDI-TOF MS system. Several phenotypic and genotypic virulence features of the staphylococcal strains were evaluated.

### 2.1. Strain identification

#### 2.1.1. Latex slide agglutination test

According to the protocol offered by the producer (Thermo Scientific™ Staphytect Plus™ Latex Agglutination Test), on a sterile slide one drop of the Staphytect Plus Control Reagent and one drop of the Staphytect Plus Test Reagent were dispensed on separate ends of the slide. With a sterile disposable loop 2–3 colonies of 20 hours culture grown on trypticase soy agar (TSA) were taken. The bacterial culture was emulsified in the Staphytect Plus Control Reagent drop and the procedure was repeated for the Staphytect Plus Test Reagent drop. The slide was gently rotated, and agglutination was noted when present in the test drop. Autoagglutination was checked in the control drop.

#### 2.1.2. Coagulase test–rabbit plasma method

According to the protocol recommended by the American Society for Microbiology and the procedure offered by the producer (Bio-Rad), freeze dried rabbit plasma was resuspended in the diluent present in the kit and was used in the following two methods [25]. Bacterial culture was 20 hours old grown on TSA at 35°C [25].

#### 2.1.3. Slide agglutination test

For each strain, on a glass slide 10 μl of sterile deionized water were dispensed in two places [25]. Three colonies were emulsified in each drop with a sterile disposable loop, and in one of the drops 3 μl of plasma were added [25]. The slide was gently moved, autoagglutination and clumping were noted if present in first 10 seconds [25].

#### 2.1.4. Tube test

For each strain, 500 μl of resuspended rabbit plasma were added in a glass tube [25]. Subsequently, one colony of bacterial culture was inoculated, and the tubes were incubated for 4 hours, at 35°C, without CO [25]. Clot formation was checked hourly by gently tilting the tube and positive results were noted. After 4 hours the tubes were incubated for next 20 hours at 25°C and clot formation was noted [25].

#### 2.1.5. VITEK

Bacterial culture grown 24 hours at 35°C on TSA were used to prepare a 0.5 McFarland (1.5 x 10^8^CFU/ml) inoculum in 3 ml of 0.45% sterile saline water. ID-GP (Gram positive identification) VITEK cards from Biomerieux were used on VITEK 2 Compact System.

#### 2.1.6. MALDI-TOF MS

Bacterial culture grown 24 hours at 35°C on TSA were placed on a stainless-steel MALDI-TOF plate with a sterile disposable loop and covered with 1 μl of Brucker matrix. The identification was done using MALDI Biotyper Compass software. Scores of ≥ 2 were taken into account. When lower scores were obtained, the procedure was repeated.

### 2.2. Phenotypic assessment of biofilm development *in vitro*

The ability to produce biofilms *in vitro*, on inert substrate, at 24, 48, and 72 hours was evaluated by violet crystal microtiter method [26]. Despite its limits this is a frequently and important screening approach [27].

One hundred fifty μl of simple broth with 15μl bacterial inoculum of McFarland 0.5 (1.5 x 10^8^CFU/ml) were added in each well of a 96 sterile wells plate [26]. The bacterial inoculum was prepared from bacterial culture of about 20 hours, previously streaked on 5% blood agar and each sample was inoculated in duplicate for each of the evaluated periods of time [26]. Every 24 hours, one plate was washed three times with 150 μl sterile saline solution (0.9% NaCl), fixed with cold methanol for five minutes, and colored with violet crystal 1% for 20 minutes [26]. The colorant was washed with distilled water and 150 μl of acetic acid 33% were added in each well [26]. For the other plates, we gently removed the mix of broth and bacterial inoculum and added 150 μl of sterile simple broth in each well [26]. At the end, all the plates were read spectrophotometrically at an absorbance of 492 nm [26]. The ability of tested strains to produce biofilms was classified according to the averaged values of the duplicates as follows: the ones with an absorbance <0.2 as weak biofilm producers, between 0.2 and 0.4 as medium producers and >0.4 as strong (well represented) biofilm producers [26, 28].

### 2.3. Phenotypic assessment of secreted virulence factors production

The secretion of virulence factors was evaluated by using a culturing dependent assay. Briefly, nutritive agar base was supplemented with various substrata and biochemical indicators to allow for the detection of particular bacterial enzymes, either directly by immediate biochemical reaction or indirectly by pH indicator color change. Microbial strains were previously grown at 37°C on nutrient agar. From the 24 hours bacterial culture a bacterial inoculum of McFarland 0.5 (1.5 x 10^8^CFU/ml) was prepared and was spot inoculated with a 10 μl sterile loop in Petri dishes with nutritive media containing the specific substratum for: haemolysins, lecithinase, lipase, caseinase, gelatinase, esculinase, DN-ase, and amylase detection [28–30]. The strains were incubated for 24 hours at 37°C, and at 25°C for the next 48 hours to allow the production and observation of specific enzymatic virulence factors; their production being evaluated at 24, 48, and 72 hours incubation [28–30]. While soluble virulence factors secreted by bacteria are mainly associated with features of invasiveness, tissue destruction, and dissemination of infection, the development of biofilms are mainly associated with the persistence of infection, resistance, and tolerance to antimicrobials and host immune defense mechanisms. The expression of these virulence features might explain the severity of the studied diseases as well as their chronicization, converting them into difficult- to- treat infections.

For haemolysin production, the strains were spotted on 5% sheep blood agar and the aspect of the haemolytic zone surrounding the spot was noted [28–30]. For lecithinase and lipase production, the strains were spotted onto 2.5% yolk agar, and respectively on 1% Tween 80 agar, and afterwards the plates were incubated at 37°C for 24 hours [28–30]. An opaque (precipitation) zone surrounding the spot showed the lecithinase, respectively lipase production; a clear area can also appear around the spot in the lecithinase test [28–30]. To evaluate the production of caseinase, the strains were streaked on nutritive agar supplemented with 15% soluble casein, precipitation surrounding the growth area indicating casein proteolysis [28, 29]. For gelatinase production, the nutritive agar was supplemented with gelatin; the positive results were considered when a precipitate formed around the colonies [28, 29]. For DN-ase production, the strains were spotted on DNA agar medium and after incubation for 24 hours at 37°C, the change of colour from light green to pink in the area surrounding the colonies was registered as a positive reaction [28, 29]. The production of the enzyme esculinase was established using a medium containing esculin and Fe^3+^ citrate. The positive result was indicated by the black colour after the enzymatic action of beta-galactosidase transforming esculin to esculethol and glucose [28, 29]. Esculethol reacts with the ferric citrate in the media to produce a dark precipitate [28, 29]. Amylase production was noted after adding iodine solution [28, 29].

**Fig 1:**
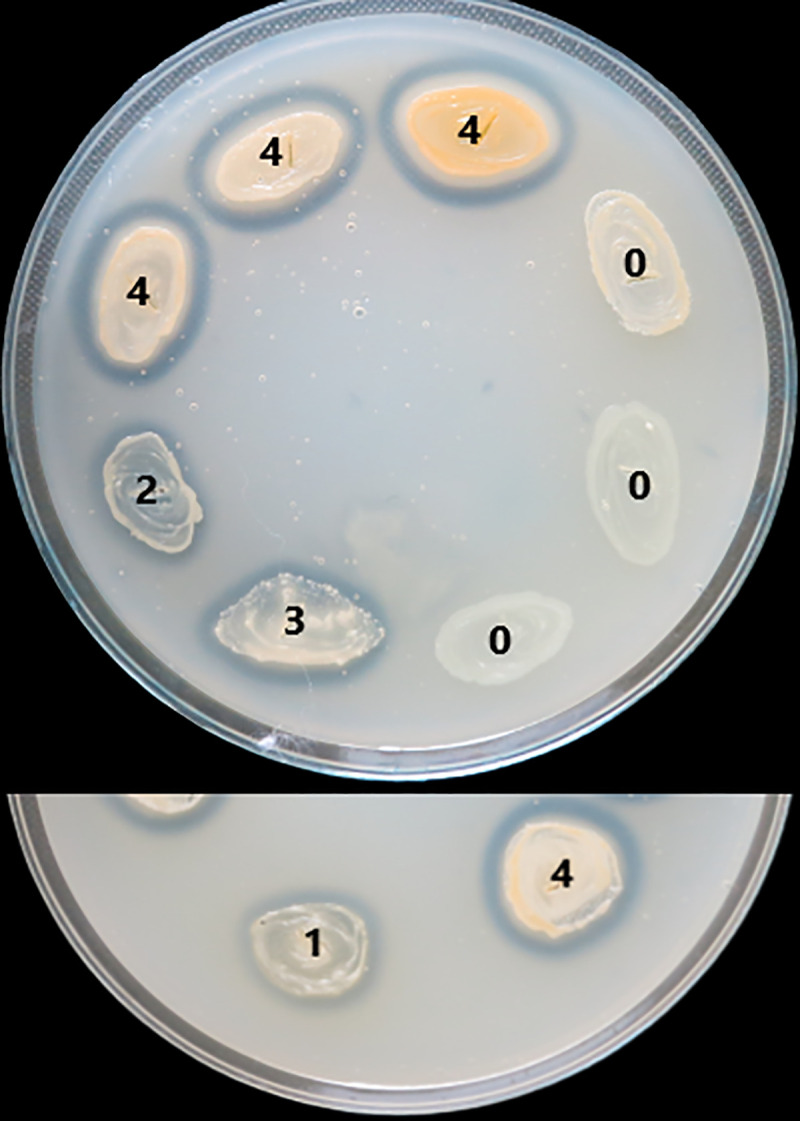
Scoring of lecithinase test according to the area of change around the culture spot.

The virulence factors production was evaluated with a score from 0 to 4 (0 was the minimum, while 4 was the maximum), depending on the diameter of the culture medium area of change around the culture spot (as exemplified in Fig 1 for lecithinase test).

### 2.4. Molecular detection of virulence genes by Polymerase Chain Reaction

Twenty-six strains (13 strains of *S*. *epidermidis*, 5 of *S*. *aureus*, 4 of *S*. *lugdunensis*, 3 of *S*. *hominis*, and 1 of *S*. *capitis*), with scores of 3 and 4 for the presence of different soluble virulence factors were selected and characterized by simplex and multiplex Polymerase Chain Reaction (PCR).

Multiple PCRs were performed in order to detect the presence of the following genes: *ebpS* (encoding elastin binding protein), *cna* (encoding collagen binding protein), *fnbA* and *fnbB* (encoding fibronectin binding proteins A and B), *fib* (encoding fibrinogen binding protein), *clfA* and *clfB* (encoding clumping factors A and B), *bbp* (encoding bone sialoprotein binding protein), *coag* (encoding coagulase), *luk-PV* (encoding Panton-Valentine leukocidin), *hlg* (encoding hemolysin), and *tst* (encoding toxic shock syndrome toxin-1; TSST-1). These sequences are conserved among various *Staphylococcus* strains and are often used to screen virulence in this genus [31, 32].

The primers used, their nucleotide sequence, and amplicon expected size can be observed in Table 1 [33].

**Table 1:** Description of primers.

Gene	Primers	Nucleotide sequence	Amplicon expected dimension (bp)	Reference
***bbp***	BBP-1	5′-AACTACATCTAGTACTCAACAACAG-3′	574	Tristan *et al*., 2003 [34]
	BBP-2	5′-ATGTGCTTGAATAACACCATCATCT-3′		
***ebpS***	EBP-1	5′-CATCCAGAACCAATCGAAGAC-3′	180	Shamsudin *et al*., 2009 [35]
	EBP-2	5′-CTTAACAGTTACATCATCATGTTTATCTTTG-3′		
***fnbB***	FNBB-1	5′-GTAACAGCTAATGGTCGAATTGATACT-3′	523	Tristan *et al*., 2003 [34]
	FNBB-2	5′-CAAGTTCGATAGGAGTACTATGTTC-3′		
***fib***	FIB-1	5′-CTACAACTACAATTGCCGTCAACAG-3′	405	Tristan *et al*., 2003 [34]
	FIB-2	5′-GCTCTTGTAAGACCATTTTCTTCAC-3′		
***clfA***	CLFA-1	5′-ATTGGCGTGGCTTCAGTGCT-3′	288	Tristan *et al*., 2003 [34]
	CLFA-2	5′-CGTTTCTTCCGTAGTTGCATTTG-3′		
***clfB***	CLFB-1	5′-ACATCAGTAATAGTAGGGGGCAAC-3′	203	Tristan *et al*., 2003 [34]
	CLFB-2	5′-TTCGCACTGTTTGTGTTTGCAC-3′		
***fnbA***	FNBA-1	5′-CACAACCAGCAAATATAG-3′	1,362	Peacock *et al*., 2002 [36]
	FNBA-2	5′-CTGTGTGGTAATCAATGTC-3′		
***cna***	CNA-1	5′-AGTGGTTACTAATACTG-3′	560	Peacock *et al*., 2002 [36]
	CNA-2	5′-CAGGATAGATTGGTTTA-3′		
***coag***	COAG-1	5′-ACCACAAGGTACTGAATCAACG-3′	812	Mullarky *et al*., 2001 [37]
	COAG-2	5′-TGCTTTCGATTGTTCGATGC-3′		
***luk-PV***	LUK-PV-1	5′-ATCATTAGGTAAAATGTCTGGACATGATCCA-3′	433	Lina *et al*., 1990 [38]
	LUK-PV-2	5′-GCATCAASTGTATTGGATAGCAAAAGC-3′		
***hlg***	HLG-1	5′-GCCAATCCGTTATTAGAAAATGC-3′	938	Lina *et al*., 1990 [38]
	HLG-2	5′-CCATAGACGTAGCAACGGAT-3′		
***tst***	TST-1	5′-CATCTACAAACGATAATATAAAGG-3'	476	Vannuffel *et al*., 1995 [39]
	TST-2	5′-CATTGTTATTTTCCAATAACCACCCG-3'		

The strains were streaked on nutrient agar and after 24 hours 1–5 colonies were added in 20 μl NaOH 0.05M plus SDS 0.25%. The tubes were put for 15 minutes at 95°C, following to add 180 μl of TE 1%. After centrifugation for 3 minutes at 13 000 rotations per minute 150 μl of the supernatant were recovered and stored at 4°C [33].

For each PCR, the prepared master mix contained 4 μl of buffer, 0.4 μl of dNTPs, 1.2 μl MgCl2, 0.1 μl Taq, 0.2 μl of each primer, and the adjusted quantity of water for a total of 19 μl, plus 1 μl of extracted DNA. The amplification used programs can be observed in Table 2.

**Table 2:** Amplification programs.

Gene	Amplification time and temperature				
	Initial denaturation	Denaturation	Annealing	Primer extension	Final extension
		Repeating cycles			
*bbp*	94°C, 5 min	25X			72°C, 10 min
*ebpS*					
*fnbB*		94°C, 1 min	55°C, 1 min	72°C, 1 min	
*fib*					
*clfA*					
*clfB*					
*fnbA*	94°C, 5 min	30X			72°C, 10 min
		94°C, 1 min	50°C, 1 min	72°C, 2 min	
*cna*	94°C, 5 min	30X			72°C, 10 min
		94°C, 1 min	55°C, 1 min	72°C, 1 min	
*coag*	94°C, 5 min	40X			72°C, 5 min
		94°C, 30 sec	55°C, 30 sec	72°C, 1.5 min	
*luk-PV*	95°C, 5 min	30X			72°C, 10 min
*hlg*					
		94°C, 30 sec	55°C, 2 min	72°C, 1 min	
*tst*	94°C, 5 min	30X			72°C, 5 min
		94°C, 30 sec	58°C, 30 sec	72°C, 2 min	

For detecting the amplicons, 5 μl of the PCR products were separated in 1.5% agarose gel.

## 3. Results

*Staphylococcus* strains were isolated from patients with skin and soft tissue difficult-to-treat infections such as severe acne vulgaris (42 cases), furunculosis (10 cases), folliculitis decalvans (21 cases), hidradenitis suppurativa (2 case), postsurgical wounds (5 cases) and other sources of purulent secretion like wound infections (7 cases) or abscesses (13 cases).

The strains were taxonomically confirmed using classical microbiological phenotypic methods and MALDI-TOFF MS system. The capacity to produce biofilm was evaluated *in vitro* by violet crystal microtiter method, while the ability to produce different soluble virulence factors was evaluated by using a culturing dependent assay on appropriate media and assessing the various effects. The presence of key virulence genes was assessed by simplex and multiplex PCRs.

### 3.1. Strain identification

**Fig 2:**
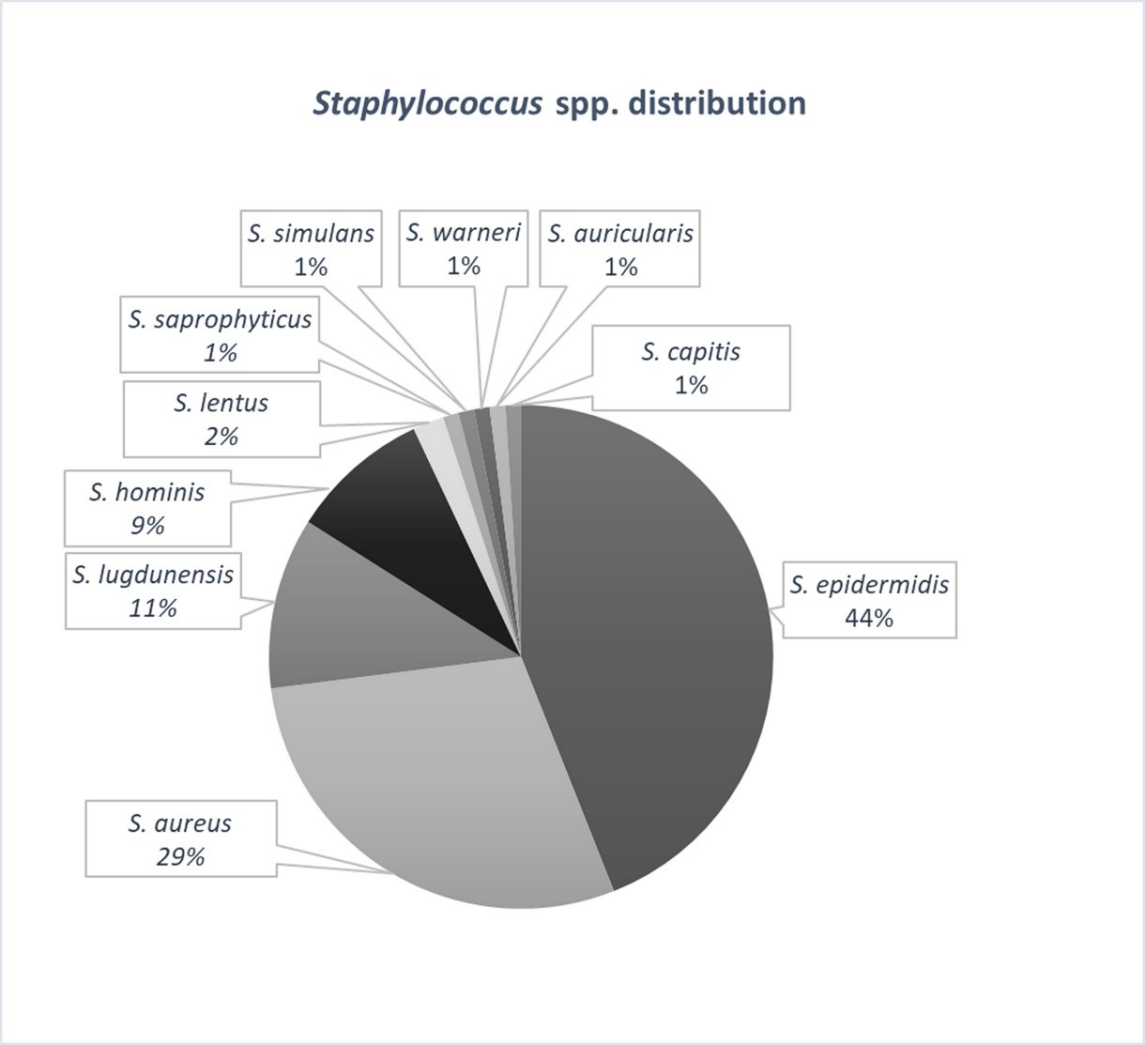
The distribution of *Staphylococcus* spp. isolated from skin and soft tissues infections.

Of the 100 isolated strains the identified species were *Staphylococcus epidermidis* (43%, 43 strains), *Staphylococcus aureus* (36%, 36 strains), *Staphylococcus lugdunensis* (9%, 9 strains), *Staphylococcus hominis* (6%, 6 strains), *Staphylococcus lentus* (2%, 2 strains), *Staphylococcus saprophyticus* (1%, one strain), *Staphylococcus simulans* (1%, one strain), *Staphylococcus warneri* (1%, one strain), and *Staphylococcus auricularis* (1%, one strain) (Fig 2). All the samples included in this study represented single patient isolates.

In what concerns the taxonomic identification the results from MALDI-TOFF MS were similar when compared with the ones obtained in VITEK testing, for only two strains of *S*. *epidermidis* VITEK identification gave two possible results–*S*. *epidermidis* or *S*. *hominis*.

### 3.2. Phenotypic assessment of coagulase production

For the 26 strains selected for molecular characterization we also performed phenotypic characterization of their capacity to produce coagulase. The characterization was performed using latex slide agglutination test, rabbit plasma slide and tube test (Table 3). For rabbit plasma tube test the results at 4 hours were identical with the ones at 24 hours, in consequence for an easier data analysis in the following table only one of the recorded results was inserted. The phenotypic production of coagulase was in accordance with the identification results performed with MALDI-TOFF and VITEK. There were only 4 special situations–for three strains (strains number 10,16 and 17) of *S*. *epidermidis* autoagglutination was observed when performing rabbit plasma slide test and one strain of *S*. *aureus* (strain number 11) was negative in the same test.

**Table 3:** Taxonomy identification and phenotypic coagulase production.

No. strain	MALDI-TOFF MS	VITEK2	Latex agglutination	Rabbit plasma slide agglutination	Rabbit plasma tube agglutination
**1**	*S*. *epidermidis*	*S*. *epidermidis*	-	-	-
**2**	*S*. *epidermidis*	*S*. *epidermidis/hominis*	-	-	-
**3**	*S*. *epidermidis*	*S*. *epidermidis*	-	-	-
**4**	*S*. *epidermidis*	*S*. *epidermidis*	-	-	-
**5**	*S*. *epidermidis*	*S*. *epidermidis*	-	-	-
**6**	*S*. *epidermidis*	*S*. *epidermidis*	-	-	-
**7**	*S*. *lugdunensis*	*S*. *lugdunensis*	-	-	-
**8**	*S*. *epidermidis*	*S*. *epidermidis*	-	-	-
**9**	*S*. *aureus*	*S*. *aureus*	+	+	+
**10**	*S*. *epidermidis*	*S*. *epidermidis/hominis*	-	Autoagglutination	-
**11**	*S*. *aureus*	*S*. *aureus*	+	-	+
**12**	*S*. *aureus*	*S*. *aureus*	+	+	+
**13**	*S*. *aureus*	*S*. *aureus*	+	+	+
**14**	*S*. *aureus*	*S*. *aureus*	+	+	+
**15**	*S*. *epidermidis*	*S*. *epidermidis*	-	-	-
**16**	*S*. *epidermidis*	*S*. *epidermidis*	-	Autoagglutination	-
**17**	*S*. *epidermidis*	*S*. *epidermidis*	-	Autoagglutination	-
**18**	*S*. *lugdunensis*	*S*. *lugdunensis*	-	-	-
**19**	*S*. *aureus*	*S*. *aureus*	+	+	+
**20**	*S*. *aureus*	*S*. *aureus*	+	+	+
**21**	*S*. *aureus*	*S*. *aureus*	+	+	+
**22**	*S*. *aureus*	*S*. *aureus*	+	+	+
**23**	*S*. *aureus*	*S*. *aureus*	+	+	+
**24**	*S*. *aureus*	*S*. *aureus*	+	+	+
**25**	*S*. *epidermidis*	*S*. *epidermidis*	-	-	-
**26**	*S*. *aureus*	*S*. *aureus*	+	+	+

### 3.3. Phenotypic characterization of biofilm development *in vitro*

**Fig 3:**
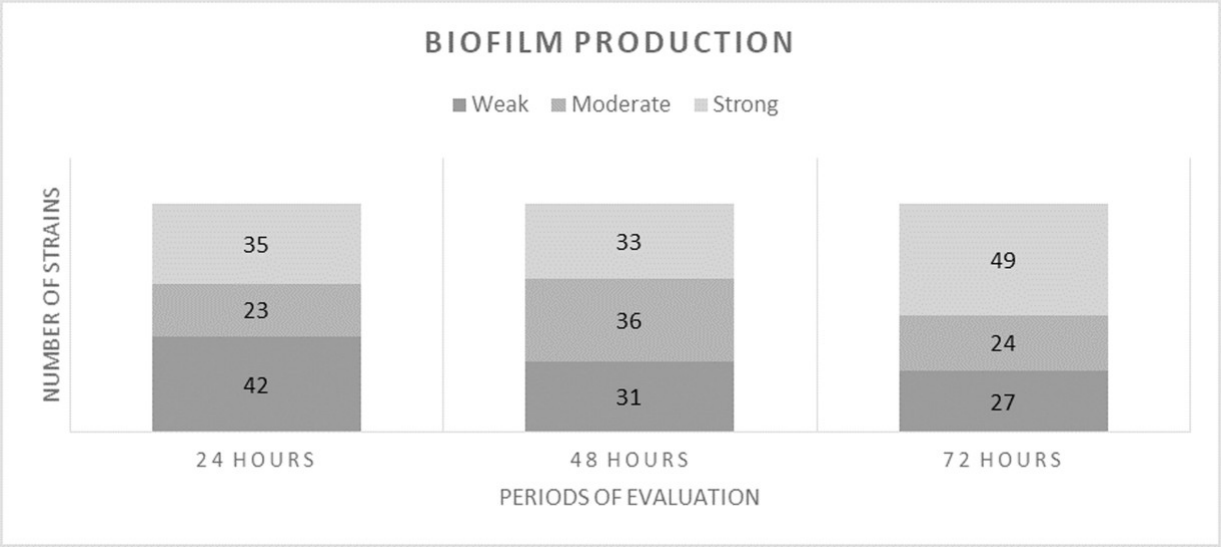
The ability of staphylococcal strains to produce biofilms (weak, moderate, strong), evaluated at 24, 48 and 72 hours.

Concerning the adherence to inert substrate and biofilm production, most of the strains were able to develop biofilms. Approximately one third (35%) presented a strong ability to develop biofilms at 24 hours, increasing to almost half of the strains (49%) at 72 hours (Fig 3). In *S*. *aureus* it was noticed a higher percentage of strains able to produce strong biofilm, at 24 hours (44,28% of the strains), 48 hours (44.83% of the strains) and 72 hours (58.62% of the strains), compared with coagulase negative strains 29.58%, 28.17%, and 45.07%.

### 3.4. Phenotypic characterization of secreted virulence factors production

Most of the strains produced caseinase (89%), lipase (71%) and lecithinase (67%). The production of haemolysins was noticed in 35% of the strains, while esculinase was identified in 27% of them. A quarter of the strains were positive for DN-ase production, 11% for gelatinase, and only 2% for amylase.

Only 2 strains were negative for the tested virulence factors, while from the positive ones (98), the majority expressed two or three of the assessed soluble virulence factors (26, respectively 28 strains).

**Fig 4:**
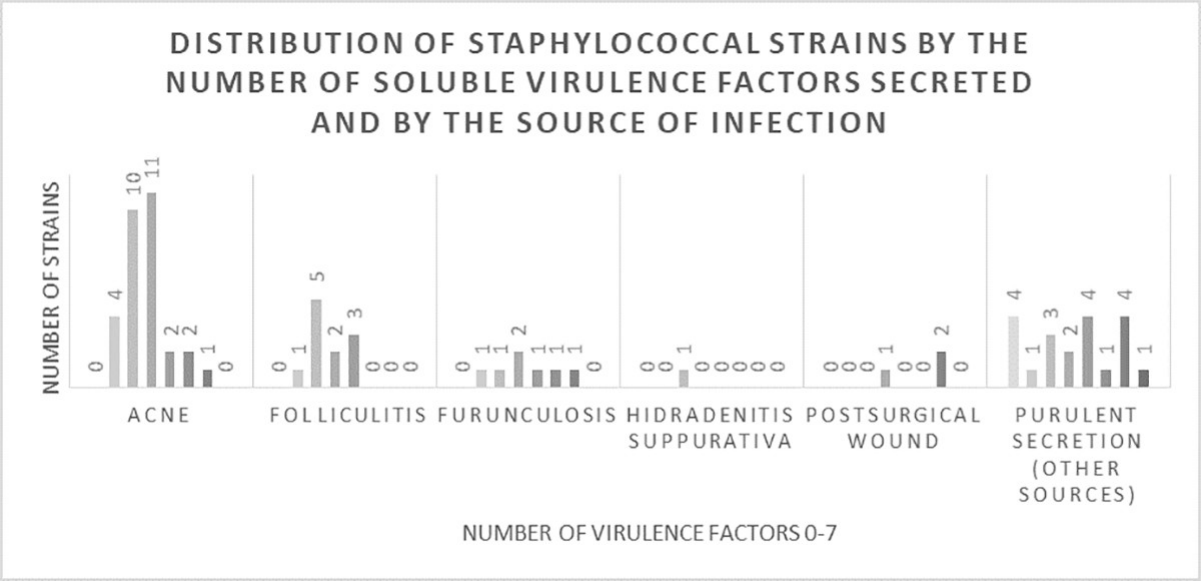
The distribution of staphylococcal strains by the number of virulence factors secreted (from none to seven) and by the source of infection. The majority of strains isolated from acne produced two or three virulence factors, while the number of virulence factors secreted by bacteria from other sources of purulent secretions was relatively homogenous.

Regarding the distribution according to the type of infection, most of the tested soluble virulence factors were identified from acne or other sources of purulent secretions. The majority of strains isolated from acne produced 2 or 3 virulence factors, while the number of virulence factors secreted by bacteria from other sources of purulent secretions was relatively homogenous. Of the coagulase-negative strains, 2.83% strains did not secreted any virulence factors, 12.68% secreted only one factor, 30.99% strains secreted 2 factors, 33.8% strains secreted 3 factors, 7.04% strains secreted 4 factors, 7.04% strains secreted 5 factors, 5.63% strains secreted 6 factors, none of the strains secreted seven virulence factors. Most of the *S*. *aureus* strains (9 strains) produced 4 virulence factors (Fig 4). The ability of these strains to produce proteases (i.e. caseinase) and pore forming toxins (i.e. lipase, lecithinase) could explain the severity of associated clinical manifestations in such infections, like the development of painful pustules, nodules and/or cysts. Moreover, we noticed an increased capacity of the strains isolated from acne or other sources of purulent secretions to produce more virulence factors, than the ones isolated from folliculitis, furunculosis, hidradenitis suppurativa or postsurgical wounds.

### 3.5. Molecular characterization of virulence genes

The molecular analysis showed that from the 26 strains, 16 of them (61.53%) harboured the *cna* gene, 15 (60%) *hlg* gene, 15 (60%) *clfB* gene, 14 (53.84%) *clfA* gene, 13 (50%) *tst* gene, 10 (38.46%) *fnbA* gene, 7 (26.92) *coag* gene, 6 (23.07%) *fnbB* gene, 5 (19.23%) *luk-PV* gene, 5 (19.23%) *fib* gene, 2 (7.69%) *bbp* gene, and none the *ebpS* gene. The taxonomy and the molecular virulence profile of each of the analyzed strains can be seen in Table 4.

**Table 4:** Virulence genes profiles of the analyzed strains (“+” and “-” indicate the presence and the absence of the gene).

No. strain	Species	*bbp*	*clfA*	*clfB*	*fib*	*fnbA*	*fnbB*	*tst*	*coag*	*cna*	*hlg*	*luk*
**1**	*S*. *epidermidis*	-	+	+	-	+	-	+	-	+	-	-
**2**	*S*. *epidermidis*	-	+	+	-	+	-	+	-	+	+	-
**3**	*S*. *epidermidis*	-	+	+	-	+	-	-	-	+	+	-
**4**	*S*. *epidermidis*	-	-	-	-	-	-	+	-	+	+	+
**5**	*S*. *epidermidis*	-	+	+	-	-	+	+	-	+	-	-
**6**	*S*. *epidermidis*	-	-	+	+	+	+	-	-	-	-	-
**7**	*S*. *lugdunensis*	-	-	-	-	-	-	-	-	+	+	+
**8**	*S*. *epidermidis*	-	-	-	-	-	-	+	-	-	-	-
**9**	*S*. *aureus*	-	+	+	+	+	-	-	+	+	+	-
**10**	*S*. *epidermidis*	-	-	-	-	-	-	+	-	+	+	-
**11**	*S*. *aureus*	-	+	+	-	-	+	+	-	+	+	-
**12**	*S*. *aureus*	-	+	+	-	-	+	+	+	+	+	-
**13**	*S*. *aureus*	-	+	+	-	+	-	+	+	-	-	-
**14**	*S*. *aureus*	-	+	+	-	+	-	-	-	-	-	-
**15**	*S*. *epidermidis*	-	-	-	-	-	-	-	-	-	-	-
**16**	*S*. *epidermidis*	-	-	-	-	-	-	-	-	-	-	-
**17**	*S*. *epidermidis*	-	-	-	-	-	-	-	-	+	+	-
**18**	*S*. *lugdunensis*	-	-	-	-	-	-	+	-	+	+	+
**19**	*S*. *aureus*	-	+	+	+	+	+	+	+	+	+	+
**20**	*S*. *aureus*	+	+	+	+	+	-	-	+	+	+	+
**21**	*S*. *aureus*	+	+	+	+	-	+	+	+	-	-	-
**22**	*S*. *aureus*	-	+	+	-	+	-	-	-	+	+	-
**23**	*S*. *aureus*	-	-	-	-	-	-	-	-	-	-	-
**24**	*S*. *aureus*	-	+	+	-	-	-	-	-	-	-	-
**25**	*S*. *epidermidis*	-	-	-	-	-	-	-	-	+	+	-
**26**	*S*. *aureus*	-	-	-	-	-	-	+	+	-	+	-

Of the 15 strains possessing the *hlg* gene, only six revealed a positive result in the phenotypic testing (3 strains of *S*. *aureus*, and 3 strains of *S*. *epidermidis*). The strains positive at PCR for the *bbp* gene (2 strains of *S*. *aureus*) or the *fib* gene (1 strain of *S*. *epidermidis* and 4 strains of *S*. *aureus*) produced a strong biofilm. For the strains possessing *clfB* gene, the biofilm production was strong for four of them (4 strains of *S*. *aureus*), moderate for five (four strains of *S*. *epidermidis* and 1 strain of *S*. *aureus*), and weak for six (5 strains of *S*. *aureus*, and 1 strain of *S*. *epidermidis*). For the presence of the *fnbA* gene, we have not observed a pattern in the biofilm production, while for the *fnbB* half of the strains produced a strong biofilm, 3 out of 6 strains, all *S*. *aureus*. In almost a third of the strains the presence of *cna* gene was associated with a strong biofilm 6 out of 16 strains (3 strains of *S*. *aureus*, 2 strains of *S*. *epidermidis*, 1 strain of *S*. *lugdunensis)*.

Most of the strains that developed a strong biofilm were isolated from purulent secretions of other sources (51.61%), a third from acne (32.25%), and less from furunculosis (6.45%), while for the ones with a weak biofilm production, acne was the main pathology (34%), followed by folliculitis (11.76%) and furunculosis (11.76%).

Two of the *S*. *aureus* strains harbored nine and ten of the 11 tested virulence genes (strains 19, 20). For other two *S*. *aureus* strains we identified a similar virulence profile consisting in the presence of 7 virulence genes (58.33%), with the exception of *coag* gene (strains 11, 12). The other strains harbored 7 different genes (63.63%) (two strains of *S*. *aureus*, number 9 and 21), 6 genes (54.54%) (one strain of *S*. *epidermidis* and one strain of *S*. *aureus*), 5 genes (45.45%) (strains 1, 3, 5, 13, 22), 4 genes (36.36%) (strains 4, 6, 7, 18), 3 genes (27.27%) (strains 10, 14, 26), 2 genes (18.18%) (strains 17, 24, 25) and 1 gene (9.09%) (strain 8). Only 3 out of the 26 analyzed strains didn’t exhibit any of the analyzed genes.

Regarding the distribution of genes for each species, the number of virulence genes identified in *S*. *epidermidis* strains varied between 0 and 6 genes. *S*. *aureus* harbored between 0 and 10 different virulence genes, and *S*. *lugdunensis* had 4 genes. *S*. *aureus* strains had a median of 5.5 genes, while for coagulase-negative staphylococci the median number of harbored genes was 4.

Approximately 20% of the strains screened for the presence of genes encoding virulence factors were *S*. *aureus*. Comparing the proportions between *S*. *aureus* and coagulase-negative staphylococci strains having the gene, a two to four times higher number of positive strains was observed among *S*. *aureus* strains for the following genes *bbp*, *clf*, *clf*, *fib*, *fnbB*, and *tst*.

## 4. Discussion

*Staphylococcus* spp. colonize a large proportion of the healthy human population, the carriage rate reported in different studies varying from 37.8% nasal carriers to 54.7% when throat sampling was included [5].

*Staphylococcus* spp. is also involved in a variety of infections, with multiple localizations and diverse evolutions [23]. The high variety of staphylococcal infections may be explained through the diversity of produced virulence factors, surface proteins, polysaccharides, enzymes, and toxins [23].

According to the results of the present study, all the strains expressed at least one virulence factor, most frequently being identified the production of caseinase, lipase, and lecithinase. Although the strains were isolated from localized infections, the identification of these factors is of great importance. The majority of the staphylococcal strains produced lecithinase, a factor that contributes to the dissemination of infection, revealing the risk of a severe evolution of an otherwise localized infection. Moreover, the production of lipase translates into the ability to convert local host tissues into nutrients vital for bacterial development and also augments the possibility to penetrate and affect host tissues [40].

The adherence to substrate is significantly increased in the case of difficult to treat infections. For example, in a study that included 103 strains of *S*. *aureus* isolated from nosocomial infections, more than half of the strains had an adherence index over 80%, most frequently in an aggregative pattern [40]. The ability to attach to an inert substrate and to develop biofilms seems to vary depending on the pathology from which the strains have been isolated (infected skin lesions, respiratory illnesses, and others). In the study of Kot B *et al*., 2018, the strongest biofilm was formed by strains originating from infected skin wounds compared to other infected organs (Eg. respiratory tract) [41]. In our study, the strains from purulent secretions (including wounds) also showed a significant ability to develop biofilms *in vitro*.

The types and severity of infections are influenced by the soluble factors produced by bacteria. For example, in some studies, the most virulent strains were isolated from blood cultures and peritoneal liquid, while in ocular infections were found bacteria with less virulent factors production [40].

Other studies from our country reported that the most frequently identified genes in *Staphylococcus* spp. were *clfA*, *clfB*, *fib*, *ebpS*, less *luk-PV* or *tst*, results similar to ours [40, 42].

Studies from other countries report a smaller percent of *tst* positive strains, like 12.8% in a study including strains collected from an Iranian hospital [43], <1% in a hospital from Tianjin [44] or none in Benin [45]. On the other side, a study from our country on strains isolated from skin infections following adverse drug reactions, found no strains harboring the *tst* gene [46].

Regarding the harboring of *fib* gene compared with the approximately 20% presence in our strains, other Romanian studies reported 14% [46]. For the presence of *bbp* gene we had similar results, but for *hlg* gene we had more than approximately 60% of the strains positive, a much higher percent than the one of 7%, reported by Gheorghe I *et al*., 2016 [46]. The importance of their presence is enhanced by the increased ability of the strains to produce a strong biofilm. Compared with our results, where more than half of the tested strains presented the *clfA* and *clfB* genes, other studies from our country showed that only 36%, respectively 37% of the strains had the genes [46].

The presence of *clfA* and *clfB*, genes that encode clumping factors have an extremely important role in the adhesion to host cells, which might influence the long term and difficult to treat infections [47]. In difficult to treat infections, most often antimicrobial therapy is not efficient, especially if the isolated strain produces multiple virulence factors, the evolution may have multiple complications [48]. Alternative therapies must be identified, for example, the anti-staphylococcal vaccine has started to regain attention [48]. Being frequently identified in staphylococcal infections, *clfA* was studied in the development of anti-staphylococcal vaccine, which administered intra-nasal in mice, reduced the risk of staphylococcal arthritis and renal abscesses, most probably thanks to a Th1 and Th17 cellular immune response [49]. In the United States of America, approximately 108 000 persons suffer annually of invasive infections with MRSA. An estimation of the administration of a staphylococcal vaccine with 40% efficiency and 64% coverage for persons over 65 years old and 35% for the rest, might reduce the number of infections by 17.6% and the number of deaths by 4,260 [47].

In four strains of *S*. *aureus* PCR testing yielded negative results for the *coag* gene (strains 11, 12, 23, 24). For the same strains phenotypic tests to determine the presence of coagulase (latex agglutination, rabbit plasma slide agglutination and rabbit plasma tube agglutination) were positive, except for a negative rapid plasma slide agglutination test in strain 11. *Coag* gene was identified only in *S*. *aureus* strains. The information from the published scientific literature on *coag* gene negative *Staphylococcus aureus* is scarce, but there exist reports of such genotypic virulence patterns [50–52]. On the other hand, there were published cases of negative phenotypic coagulase tests in *S*. *aureus* strains [53, 54]. It was also mentioned the possibility that otherwise considered coagulase negative staphylococci, such as *S*. *lugdunensis* and *S*. *schleiferi subsp*.*schleiferi*, may produce clumping factor [51, 55]. These results may be due to coagulase gene polymorphism [56], to mutations of the *coag* gene in the regions of the primers used for molecular tests, the presence of other factors that may lead to coagulase positive phenotypic tests, such as other clumping factors coded by *clfA* or *clfB* (both positive in strains 11,12, 24) [51, 57], or others. We aim to perform a systematic review on the subject, to discuss the sensitivity and specificity of different laboratory tests and debate the gold standard use of PCR in differentiating coagulase negative and positive staphylococci, as well as to conduct a future study to identify phenotypically and genotypically the coagulase virulence factor in a statistically relevant number of *S*. *aureus* strains.

## 5. Conclusions

Resistance and tolerance to antimicrobials represent great obstacles in treating staphylococcal infections, since it was observed that in some cases, although the strains were susceptible to the majority of available antimicrobial drugs, the evolution was severe and with limited response to treatment. In these cases, one of the possible explanations might be the virulence profile of the strains. We have analyzed 100 *Staphylococcus* spp. strains obtained from various wounds and identified a wide range of virulence factors, such as proteases, pore forming toxins, lipases and DN-ases. The most frequently described virulence features were the protease caseinase and the pore forming toxin lipase, and almost half of the strains were able to produce a strong biofilm after 72 hours incubation in standard, static conditions. Most often we identified the following virulence genes: *cna*, *hlg*, *clfB*, *clfA*, *tst*, *fnbA* and *coag*, and for the strains possessing the *fnbB*, *can*, *bbp* and *fib* gene we noticed a higher biofilm development capacity. The identified factors are well known for their ability to enhance the bacterial attachment, penetration in the affected tissue and dissemination, increasing the risk of severe evolution of infection. Strains isolated from purulent secretions were higher biofilm producers, results supported also by the molecular analysis, for which the most often identified genes were those involved in adhesion. Moreover, we observed that the strains isolated from acne produced more frequent the analyzed virulence factors.

These results support the idea that virulence is a key element in the development of the infectious process and understanding the diversity and complexity of virulence modulation represents one of the major alternative strategies for new and personalized therapeutic approaches.
